# INSPEcT-GUI Reveals the Impact of the Kinetic Rates of RNA Synthesis, Processing, and Degradation, on Premature and Mature RNA Species

**DOI:** 10.3389/fgene.2020.00759

**Published:** 2020-07-17

**Authors:** Stefano de Pretis, Mattia Furlan, Mattia Pelizzola

**Affiliations:** Center for Genomic Science of IIT@SEMM, Fondazione Istituto Italiano di Tecnologia, Milan, Italy

**Keywords:** transcription, mathematical modeling, graphical user interface (GUI), RNA synthesis, RNA processing, RNA degradation

## Abstract

The abundance of RNA species and their response to perturbations are set by the kinetics rates of RNA synthesis, processing, and degradation. However, the visualization, interpretation, and manipulation of these data require familiarity with mathematical modeling and command line tools. INSPEcT-GUI is an R-Shiny interface that allows researchers without specific training to effortlessly explore how the fine kinetic regulation of the RNA life cycle can shape gene expression programs. In particular, it allows to: (i) interactively visualize gene-level RNA dynamics; (ii) refine the model fit of experimental data; (iii) test alternative regulatory models; (iv) explore, independently from the availability of data, how the combined action of the RNA kinetic rates impacts on premature and mature RNA. INSPEcT-GUI is freely available within the R/Bioconductor package INSPEcT at http://bioconductor.org/packages/INSPEcT/. An HTML vignette including documentation on the tool startup and usage, executable examples, and a video demonstration, are available at: http://bioconductor.org/packages/release/bioc/vignettes/INSPEcT/inst/doc/INSPEcT_GUI.html.

## Introduction

The RNA life-cycle is composed by three main steps - the synthesis of premature RNA, its processing into the mature form, and the degradation of the latter. The dynamics of transcripts metabolism are set by the rates governing the kinetics of those steps, ultimately setting the abundance of premature and mature RNA species (Shalem et al., 2008; Friedel et al., 2009; Rabani et al., 2011; Eser et al., 2013; de Pretis et al., 2017), and shaping their temporal response following a perturbation (Zeisel et al., 2011). The dynamics of a given transcript are described by a system of ordinary differential equations, which includes the abundance of RNA species (*P* and *M*, the concentrations of premature and mature RNA) and the RNA kinetic rates (*k*_1–3_, the rates of RNA synthesis, processing, and degradation):

(1) $$\begin{array}{c} \frac{d\,P}{d\,t}=k_{1}-k_{2}\cdot P\\ \frac{d\,M}{d\,t}=k_{2}\cdot {\text{P - k}}_{3}\cdot M \end{array}$$

This model is implemented, with various assumptions, by different tools [cDTA (Sun et al., 2012), DRiLL (Rabani et al., 2014), INSPEcT (de Pretis et al., 2015) and pulseR (Uvarovskii and Dieterich, 2017)], which rely on the quantification of both nascent and total RNA species, the former profiled through RNA metabolic labeling (Dolken et al., 2008). Recently, novel approaches are being developed that do not require the quantification of nascent RNA, to estimate the full set (Furlan et al., 2019), or a subset of the kinetic rates (Zeisel et al., 2011; Gray et al., 2014; La Manno et al., 2018).

Despite the availability of these tools, anticipating the outcome of the joint contribution of various RNA life-cycle stages can be far from trivial. For example, how would a halved RNA degradation impact on the expression of a gene? How would the latter be affected if also the rate of RNA processing were increased? A tool that enable scientists to explore and manipulate these data, while not requiring specific training on mathematical modeling or familiarity with command line scripting, is missing. To lower the barrier to the field, we developed INSPEcT-GUI, an R-Shiny interface that fully complements the analytical framework implemented in INSPEcT (de Pretis et al., 2015). INSPEcT-GUI greatly facilitates the visualization, interpretation and manipulation of the RNA dynamics of individual genes, and allows the user to explore *de novo* the role of the RNA kinetic rates.

## Implementation

The RNA kinetic rates (synthesis, processing, and degradation) are modeled within INSPEcT-GUI with the following analytical functions: constant, sigmoid, or impulse. Thus, one of these functions, and the corresponding parameters, has to be set for each rate.

**FIGURE 1:**
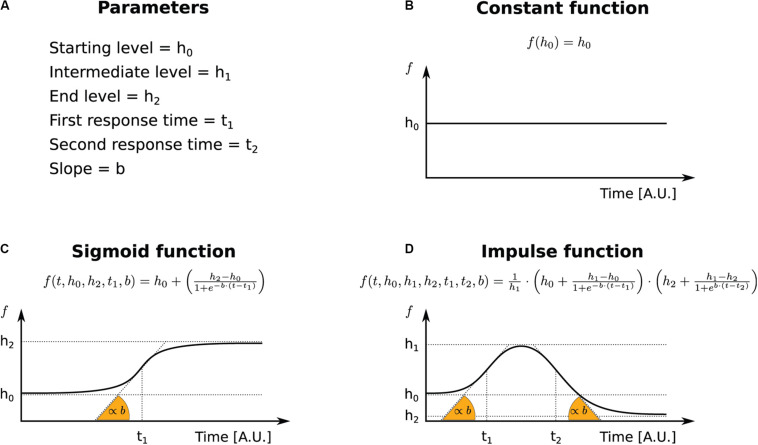
The functional forms used to parameterize transcriptional and post-transcriptional responses. **(A)** Legend for the terms used in the INSPEcT-GUI to identify function’s parameters and the corresponding symbols in the figure. **(B)** Constant function. **(C)** Sigmoid function. **(D)** Impulse function.

At steady state, constant functions constrain the kinetic rates to fixed values (Figures 1A,B), determining how a given combination of rates set the steady-state abundance of RNA species. Thus, the system in Eq. (1) reduces to:

(2) $$\begin{array}{c} P=\frac{k_{1}}{k_{2}}\\ M=\frac{k_{1}}{k_{3}} \end{array}$$

Rather, when at least one of the three kinetic rates is set to variable, the abundance of premature and mature RNA species is determined through the numerical solution of the system of ordinary differential equations indicated in Eq. (1). Variable rates can be described by sigmoid or impulse functions. Sigmoids are S-shaped functions described by four parameters: starting and final levels, time of transition between those, and slope of the response (Figure 1C). Impulse functions allow a more complex behavior, with two additional parameters that describe time and levels of a second transition, possibly originating double sigmoids or bell-shaped responses (Chechik and Koller, 2009) (Figure 1D).

**FIGURE 2:**
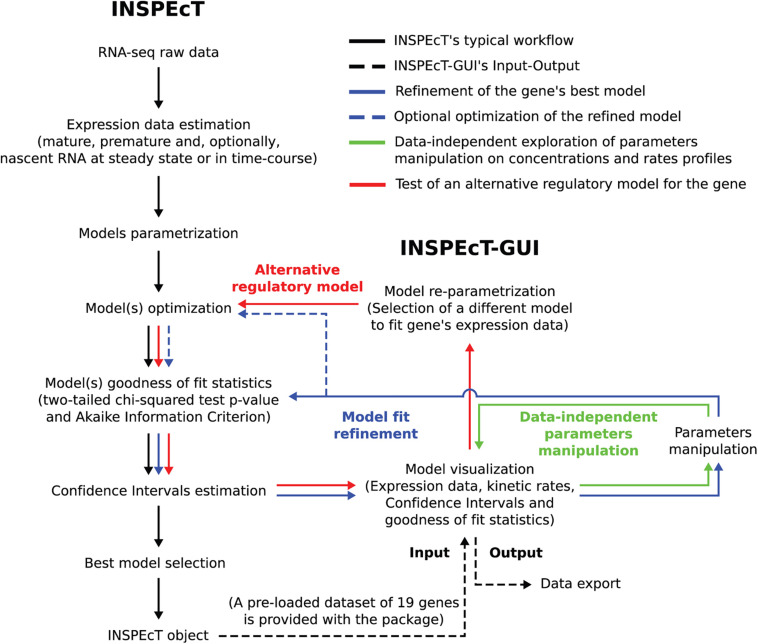
Flowchart illustrating how INSPEcT-GUI integrates with and extend the functionalities implemented in INSPEcT. Black arrows trace the typical INSPEcT pipeline, from raw RNA-seq data to an INSPEcT object containing, for each gene in the dataset, the modeled kinetic rates together with the goodness of fit statistics. Dashed black arrows depict the Input of an INSPEcT object to INSPEcT-GUI and the output of the latter. Green arrows indicates how the GUI can be used, without the need of any data, for the manipulation of model’s parameters to observe the effect on the profiles of RNA species and kinetic rates. Solid blue arrows indicate how the GUI can be used to manipulate a model’s parameters, thus refining the INSPEcT model through the reassessment of goodness of fit statistics and confidence intervals. Dashed blue arrows indicate how also the numerical optimization can optionally be updated. Red arrows indicate how the GUI can be used to implement an alternative regulatory model (i.e., switching between the functions of Figure 1 for one of more rates), which is subsequently optimized and tested.

When INSPEcT-GUI is applied to RNA expression data, it infers the parameters of the functional forms assigned to *k*_1–3_. In the “user defined” mode the parameters of *k*_1–3_ functions can be freely assigned to model RNA kinetic rates and explore their impact on premature and mature RNAs. Figure 2 illustrates the analysis and statistical framework of INSPEcT, and depicts how INSPEcT-GUI integrates with and extends the functionalities implemented in INSPEcT.

### Visualizing and Manipulating RNA Dynamics Based on Experimental Data

**FIGURE 3:**
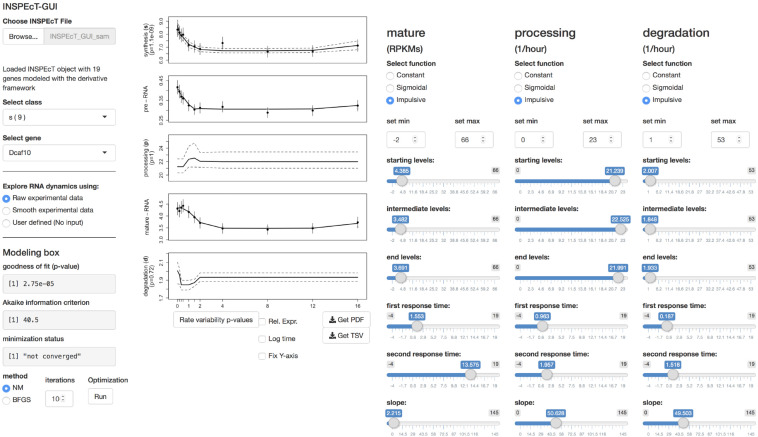
The interface of INSPEcT-GUI. The upper left part controls the upload of an INSPEcT dataset, generated with or without the profiling of nascent RNA, and allows selecting a gene with a given regulatory model. In the lower left part, the user can choose whether to plot raw or smoothed data, and modeling statistics are reported and updated in real time in case of changes. In the central part, the gene-level abundance of RNA species and the RNA kinetic rates are displayed. On the right most part, the control panel allows setting the fitting of the model with specific functions.

INSPEcT modeling results for both steady state or time-course experiments can be uploaded within INSPEcT-GUI for an interactive visualization and for further analyses (Figure 3). To facilitate the generation of the dataset, a wrapper function has now been included in INSPEcT that allows running the tool with one single command, starting from BAM files or a single-gene PCR data. The INSPEcT object returned by the wrapper is ready to be imported in INSPEcT-GUI. Notably, INSPEcT can quantify the RNA kinetic rates without requiring the profiling of nascent RNA (Furlan et al., 2019), and these datasets are fully supported by INSPEcT-GUI.

INSPEcT-GUI allows controlling the INSPEcT analysis framework to reassess and potentially refine the selected regulatory model. Analyzed genes are divided according to their modeled RNA dynamics, grouping together those that have the same regulatory model. For example, “sd” stays for variable synthesis, constant processing, and variable degradation rates, while “sp” stays for variable synthesis, variable processing, and constant degradation rates. Once a gene is selected, the temporal or steady state profiles of premature and mature RNA species are plotted, together with the corresponding profiles of the kinetic rates.

All plots include standard deviation and/or 95% confidence intervals, and experimental data can be smoothed. The functional forms assigned to each kinetic rate, and the corresponding parameter settings, are reported. Moreover, goodness of fit statistics are indicated, expressed as two-tailed chi-squared test *p*-value and Akaike information criterion. These metrics are penalized for the model complexity, and can be used for the comparative evaluation of alternative regulatory models (Browne and Cudeck, 2016). Moreover, the chi-squared test *p*-value can be used to assess whether the model under consideration adequately explains the data. These metrics are evaluated in real-time, helping the user assessing if a change in the model parameters is lowering or increasing the ability to interpret the data.

The model fit can be reassessed and potentially improved by providing additional minimization iterations, or by selecting a different minimization algorithm [choosing among Nelder-Mead (Lagarias et al., 2006) and BFGS (Browne and Cudeck, 2016)].

Finally, alternative regulatory models can be tested by changing the functional forms assigned to one or more kinetic rates, and/or by modifying the corresponding parameters. Upon changes of functional forms or their parameters, the corresponding plots and statistics are updated on real-time. This allows the user to evaluate the impact and goodness of fit of alternative regulatory models, without the necessity of explicitly controlling the corresponding INSPEcT functionalities, which are automatically exploited under the hood.

### Exploring RNA Dynamics Without Experimental Data: INSPEcT-GUI Simulations

The previous section illustrated how INSPEcT-GUI can take advantage of the INSPEcT analytical framework to explore or manipulate RNA dynamics of individual genes. Alternatively, INSPEcT-GUI can be used to simulate how a given combination of constant or variable RNA kinetic rates impact on the temporal profiles of premature and mature RNAs. This approach allows exploring the effect of complex transcriptional and post-transcriptional regulations, without the need of experimental data.

**FIGURE 4:**
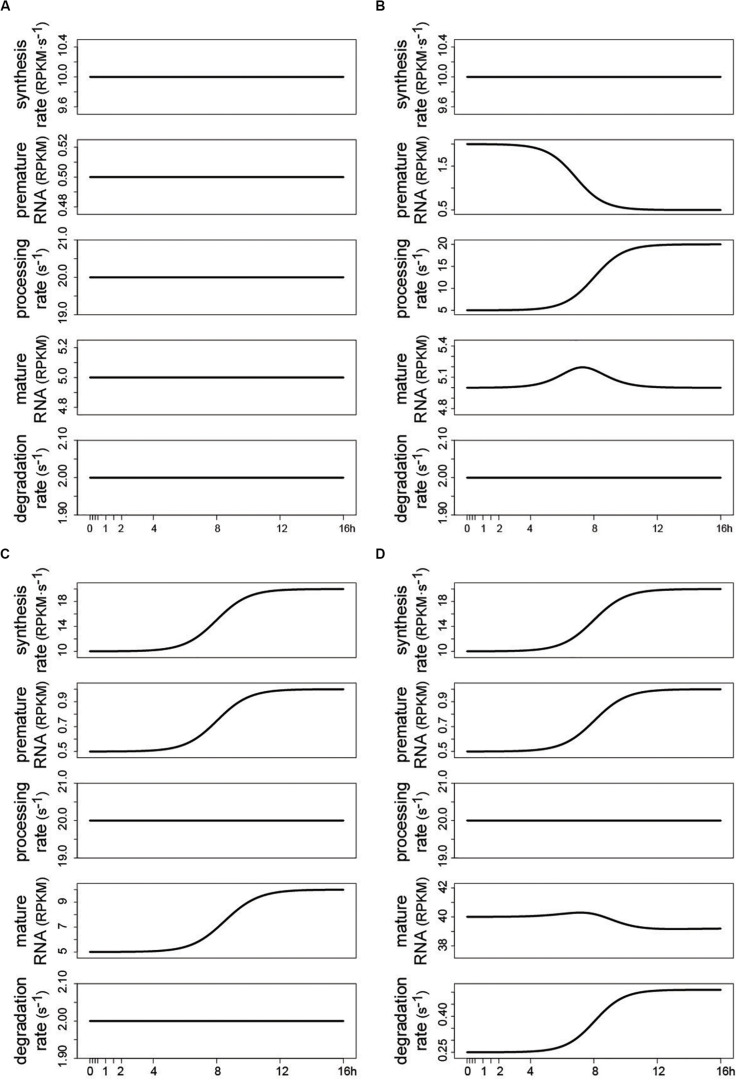
Examples of temporal RNA dynamics produced by INSPEcT-GUI in the “user defined” mode. When INSPEcT-GUI is run in the “user defined” mode, the impact of the RNA kinetic rates can be evaluated without the need of experimental data. Four examples are reported. **(A)** At steady-state constant RNA kinetic rates set the abundance of RNA species, according to Eq. (2) (see text). **(B)** Given a constant rate of synthesis, premature RNA is reduced over time due to an increase in the rate of RNA processing. **(C)** Given a constant rate of RNA processing, both premature and mature RNA species are increased over time due to an increase in the rate of RNA synthesis. **(D)** As in **(C)** but concomitant with an increase in the rate of RNA degradation. As a result, the contrasting modulation of synthesis and degradation rates levels the temporal profile of mature RNA.

Using the same interface described in the previous section, and depicted in Figure 3, a control panel allows defining the function and setting the parameters for each kinetic rate. The results are updated in real time. Few output examples are reported here, illustrating that predicting the temporal pattern of the RNA species following a change in the kinetic rates is often non trivial, as it depends on both the rates’ absolute value, and the magnitude and shape of their modulation (Furlan et al., 2019). Constant kinetic rates determine flat temporal profiles of premature and mature RNA, whose abundance is set according to the magnitude of the kinetic rates [Eq. (2); Figure 4A]. Figure 4B illustrates how the modulation of premature RNA can be obtained independently from the transcriptional input, i.e., despite a constant rate of RNA synthesis. Indeed, an increasing rate of processing leads to lower level of premature RNA. Noteworthy, mature RNA is only transiently affected by the permanent change in processing dynamics. Figure 4C shows how the modulation of the synthesis rate affects both premature and mature RNA forms. Finally, Figure 4D illustrates how the joint change of synthesis and degradation rates, if modulated in the same direction, can lead to invariant levels of mature RNA, while premature RNA abundance is permanently increased. Various regulatory scenarios, originated through the joint modulation of the kinetic rates, are illustrated in detail in the online vignette of INSPEcT-GUI, and in the included video demonstration.

## Discussion

The recent development of experimental and computational methods able to dissect the dynamics of RNA metabolism is allowing the study of layers of regulation that were previously hard to characterize, which are revealing the detailed molecular mechanisms at the basis of complex gene expression programs. INSPEcT-GUI is seamlessly integrated with INSPEcT modeling functionalities, covering steady state or time course studies with or without the profiling of nascent RNA. Altogether, INSPEcT-GUI facilitates the interactive visualization and manipulation of gene level RNA dynamics, empowering scientists with no specific training with the possibility of exploring the effects of the combinatorial regulation of RNA kinetic rates.

## Data Availability Statement

INSPEcT-GUI is freely available within the R/Bioconductor package INSPEcT at http://bioconductor.org/packages/INSPEcT/. An HTML vignette including documentation on the tool startup and usage, executable examples, and a video demonstration, are available at: http://bioconductor.org/packages/release/bioc/vignettes/INSPEcT/inst/doc/INSPEcT_GUI.html.

## Author Contributions

SP and MP conceived the study. SP and MF developed the software. All authors contributed discussing and writing the manuscript.

## Conflict of Interest

The authors declare that the research was conducted in the absence of any commercial or financial relationships that could be construed as a potential conflict of interest.
